# A chromosome-level genome of the Nicobar pigeon, *Caloenas nicobarica*

**DOI:** 10.1093/jhered/esaf031

**Published:** 2025-05-15

**Authors:** Nicolas Alexandre, Jennifer Balacco, Tatiana Tilley, Linelle Abueg, Nadolina Brajuka, Lucas R Moreira, Conor Whelan, Erika Schwarz Taylor, Erich D Jarvis, Olivier Fedrigo, Beth Shapiro, Anna L Keyte

**Affiliations:** Colossal Biosciences Inc., Dallas, TX, United States; Vertebrate Genome Laboratory, The Rockefeller University, New York, NY, United States; Vertebrate Genome Laboratory, The Rockefeller University, New York, NY, United States; Vertebrate Genome Laboratory, The Rockefeller University, New York, NY, United States; Vertebrate Genome Laboratory, The Rockefeller University, New York, NY, United States; Colossal Biosciences Inc., Dallas, TX, United States; Vertebrate Genome Laboratory, The Rockefeller University, New York, NY, United States; Form Bio Inc., Austin, TX, United States; Vertebrate Genome Laboratory, The Rockefeller University, New York, NY, United States; Howard Hughes Medical Institute, Chevy Chase, MD, United States; Colossal Biosciences Inc., Dallas, TX, United States; Colossal Biosciences Inc., Dallas, TX, United States; Colossal Biosciences Inc., Dallas, TX, United States

**Keywords:** *Caloenas nicobarica*, chromosome-level genome, Columbidae, conservation, Nicobar pigeon

## Abstract

The Nicobar pigeon (*Caloenas nicobarica*), the closest living relative of the extinct Dodo (*Raphus cucullatus*), is endemic to Southeast Asia with a fragmented distribution across numerous small islands. It suffers from habitat loss, hunting, and predation from invasive species, resulting in its classification as Near Threatened by the International Union for the Conservation of Nature. We have generated a haplotype-resolved and chromosome-level genome assembly of the Nicobar pigeon using a combination of PacBio HiFi long-read sequencing and Arima Hi-C chromatin interaction mapping. This assembly includes two haplotypes, each spanning approximately 1.2 Gb. Haplotype 1 has a contig N50 of 25.2 Mb and a scaffold N50 of 79.7 Mb, whereas haplotype 2 has a contig N50 of 24.7 Mb and a scaffold N50 of 107.9 Mb. As the first high-quality genome assembly of any bird in the Columbidae Indo-Pacific clade, this resource provides valuable insights for phylogenetic studies. Furthermore, the phylogenetic proximity of the Nicobar pigeon to the Dodo (*R. cucullatus*) and the Rodrigues Solitaire (*Pezophaps solitaria*) offers a unique opportunity to study these extinct species, making this assembly a critical resource for evolutionary studies. It also offers a unique model for studying genetic diversity, adaptation, and speciation in island environments. This genomic resource will not only enhance our understanding of the evolutionary history of the Nicobar pigeon but also serve as a valuable tool for future conservation efforts aimed at preserving this unique species and its fragile island ecosystem.

## Introduction

The Nicobar pigeon (*Caloenas nicobarica*) is a large (~38 cm, ~500 g) and strikingly iridescent bird, renowned for its blue-green plumage (Gibbs and Eustace 2000; Rashid et al. 2018). This species forms large flocks and engages in island hopping, behaviors that contribute to its broad distribution across the Indo-Pacific, where two subspecies are recognized (Baptista et al. 2020). The Nicobar pigeon is a small-island specialist with a highly fragmented distribution such that the more widespread subspecies, *C. n. nicobarica*, inhabits small and isolated groups of islands from the Andaman and Nicobar archipelago to the Solomon Islands (Putra et al. 2021). In contrast, *C. n. pelewensis* is only found in Palau. These features make it an excellent model for studying genetic diversity and biogeography on islands.

The Nicobar pigeon faces significant threats from habitat loss, hunting, and invasive species such as cats and rats, which feed on nests of breeding birds (Ali 2010). It is classified as Near Threatened by the International Union for Conservation of Nature, with local vulnerabilities amplified by habitat degradation, predation from invasive mammals, and an insular distribution. For example, the Palau subspecies has an estimated population of only ~1,000 birds. Additionally, this species is hunted extensively for food, the pet trade, and its gizzard stone, which is valued for jewelry. Understanding its genetic diversity is critical for guiding conservation efforts, particularly at the population level.

A member of the Columbidae family, the Nicobar pigeon is also the closest living relative to the extinct dodo (*Raphus cucullatus*) and the Rodrigues solitaire (*Pezophaps solitaria*), as well as the last surviving species of its genus (*Caloenas*) ( Shapiro et al. 2002; Soares et al., 2016). This phylogenetic position makes it a valuable model for studying the genetic changes underlying traits such as flightlessness, large body size, and unique beak morphology in these extinct relatives. Moreover, within the Columbidae family, the Nicobar pigeon belongs to the Indo-Pacific clade, an insular radiation that provides critical insights into the genomic basis of speciation and adaptation in island species (Soares et al. 2016).

In this study, we present a high-quality, chromosome-level genome assembly of *C. nicobari*ca. This resource will enable robust comparative genomic analyses across avian species and offer tools for understanding the evolutionary history of the Indo-Pacific clade. Importantly, this assembly will support conservation efforts by providing insights into the genetic diversity of the Nicobar pigeon, guiding strategies to mitigate population declines and preserve this unique species.

## Methods

### Biological materials

Samples were provided by the Wildlife Conservation Society and were collected on 5 May 2023 by Charles Alex at the Bronx Zoo in New York. The specimen was a juvenile male born in captivity on 7 March 2023 and registered under studbook number B23044. The bird was euthanized for medical reasons, and blood and muscle samples were collected. The blood samples were collected in two heparin tubes (2X), and stored at −80 °C. The muscle tissue was collected and immediately frozen, and stored at −80 °C.

### Nucleic acid library preparation and sequencing

We extracted high molecular weight (HMW) DNA from 20 µl of whole blood using the Qiagen HMW DNA MagAttract Kit (Qiagen 67563), following the manufacturer’s protocol. HMW DNA was quantified using the Qubit 2.0 (Invitrogen Qubit dsDNA Broad Range Assay Cat. No. Q32850), and quality was assessed with the Agilent Femto Pulse system.

For PacBio HiFi library preparation, we used 2 µg of HMW DNA. DNA fragmentation was performed with a Megaruptor 3 (Diagenode, Denville, NJ, USA) using a standard hydropore (Cat. No. E07010003) with the protocol set to speed 28. We prepared the library using the PacBio SMRTbell Prep Kit 3.0 (PN: 102-182-700) with barcoded adapters (Barcoded Overhang Adapter Kit 8B; PN: 101-628-500). Size selection was performed using Sage Science Pippin HT with the 10 kb setting to ensure the libraries contained the desired insert size range. We assessed library quantity and quality with the Agilent Femto Pulse and the Invitrogen Qubit 1X dsDNA High Sensitivity (HS) Assay Kit. The final library was sequenced on one PacBio Revio SMRT cell.

We generated a Hi-C library from 15 µl of whole blood using the Arima v2 enzymes and Arima library prep kit, following the manufacturer’s protocol. Briefly, the sample was cross-linked, and proximity ligation was performed. Fragments were then size-selected, and biotin-labeled DNA was enriched with streptavidin beads, and a final Illumina-compatible library was generated. Library quality and quantity were assessed using an Agilent Fragment Analyzer and Invitrogen Qubit 1X dsDNA HS Assay Kit. We sequenced the Hi-C library on the Illumina NovaSeq 6000 and NextSeq 500 instruments (150 bp paired-end reads).

We isolated total RNA from muscle tissue using the Qiagen RNeasy Mini kit. The RNA-seq library was prepared using the NEBNext Single Cell/Low Input cDNA Synthesis & Amplification Module (PN: NEB 6421S) and sequenced on the Illumina NovaSeq 6000 (100 bp PE). We prepared the Iso-Seq library using the SMRTbell prep kit 3.0 PacBio (PN: 102-182-700) and sequenced on one Sequel IIe SMRT cell. Full-length reads were identified, clustered, and collapsed into nonredundant transcripts using the Iso-Seq analysis pipeline in SMRT Link v12.0. This transcriptome data was used to annotate the bCalNic1.hap1 assembly through the NCBI Eukaryotic Genome Annotation Pipeline v10.2.

### Genome assembly

We performed nuclear genome assembly using the Vertebrate Genomes Project v.20 Galaxy assembly pipeline (Rhie et al. 2021; Larivière et al. 2023) (Table 1). Briefly, PacBio reads with remaining adapters were removed using Cutadapt 4.8 (Martin 2011). PacBio HiFi data were assembled into phased contigs using hifiasm with Hi-C mode (Cheng et al. 2022). The contig assembly was performed using the following parameters: homozygous read coverage (-hom-cov 73); maximum probing distance for bubble popping when generating primary/alternate contig graphs (-m 10,000,000); maximum probing distance for bubble popping when generating haplotype-resolved processed unitig graph without small bubbles (-p 100,000); small unitig size (-n 3); maximum and minimum overlap drop ratio (-x 0.8 -y 0.2); number of rounds for assembly graph cleaning (-a 4); and misjoined unitig detection enabled (--l-msjoin 500,000). Each haplotype was scaffolded with Hi-C data using YaHS (Zhou et al. 2023) with contig error correction disabled (--no-contig-ec) and Arima v2 restriction sites (-e GATC,GANTC,CTNAG,TTAA). The quality of the two draft assemblies was evaluated through a combination of metrics, such as contiguity, completeness, and base accuracy from Busco 5.7.1 (Manni et al. 2021), gfastats v1.3.6 (Formenti et al. 2022), and Merqury (Rhie et al. 2020). The mitochondrial genome was assembled separately using MitoHifi version 3 (Uliano-Silva et al. 2023).

**Table 1.** Quality metrics for genome assembly, Hi-C contact maps, organelle assembly, and genome quality.

	Software	Version
**Assembly**	VGP pipeline	V. 2.0, hifiasm-HiC on Galaxy
PacBio adapters removal	cutadapt	4.8 + galaxy0
k-mer counting	Meryl (k = 21)	1.3 + galaxy4
De novo assembly (contiging)	hifiasm	0.19.3 + galaxy0
Hi-C scaffolding	yahs	1.2a.2 + galaxy1
Estimation of genome size and heterozygosity	GenomeScope2	2.0
**Hi-C contact map**		
Alignment	bwa_mem	2.2.1 + galaxy1
Data processing	bellerophon	1.0 + galaxy1
Contact map visualization	PretextMap	0.1.9
	PretextView	0.2.5
	PretextSnapshot	0.0.4
**Organelle assembly**		
Mitogenome assembly	MitoHiFi	3 + galaxy0
**Genome quality assessment**		
Basic assembly metrics	gfstats	1.3.6
Assembly completeness	Busco (-l aves_odb10 -m genome)	5.7.1
	Merqury	1.3

We mapped Hi-C data to each assembly using BWA-MEM2 and the Arima mapping pipeline, allowing for multimapping (https://github.com/VGP/vgp-assembly/blob/master/pipeline/salsa/arima_mapping_pipeline.sh). A contact map was generated with PretextMap (https://github.com/sanger-tol/PretextMap) and visualized with PretextView (https://github.com/sanger-tol/PretextView) (Table 1). Finally, we performed an in-depth analysis of discordances in the assembly and curated the final assembly following the procedure outlined by Howe et al. (Howe et al. 2021).

We mapped PacBio reads to the final assembly using minimap2 (Li 2018, 2021). Coverage, G-C content density, and gene density were calculated using samtools 1.19.2 (Li et al. 2009) and bedtools v2.30.0 (Quinlan and Hall 2010). We also generated gene density and G-C density tracks. We used repeat regions as determined by NCBI by identifying soft masked regions and applied a sliding window approach (500 kb) to these densities and a Circos plot as generated using the R package Cirlize (Gu et al. 2014).

To generate a synteny plot, we aligned the Nicobar pigeon haplotype 1 assembly to the publicly available rock pigeon genome (*Columba livia*; GCF_036013475.1 [bColLiv1.pat.W.v2]) using minimap2 (Li 2018, 2021). Secondary alignments and synteny links shorter than 100 kb were excluded. We also ran dnadiff from the MUMmer4 package (Marçais et al. 2018) for alignment support. A synteny plot was produced using a custom R script.

## Results

**Fig. 1.**
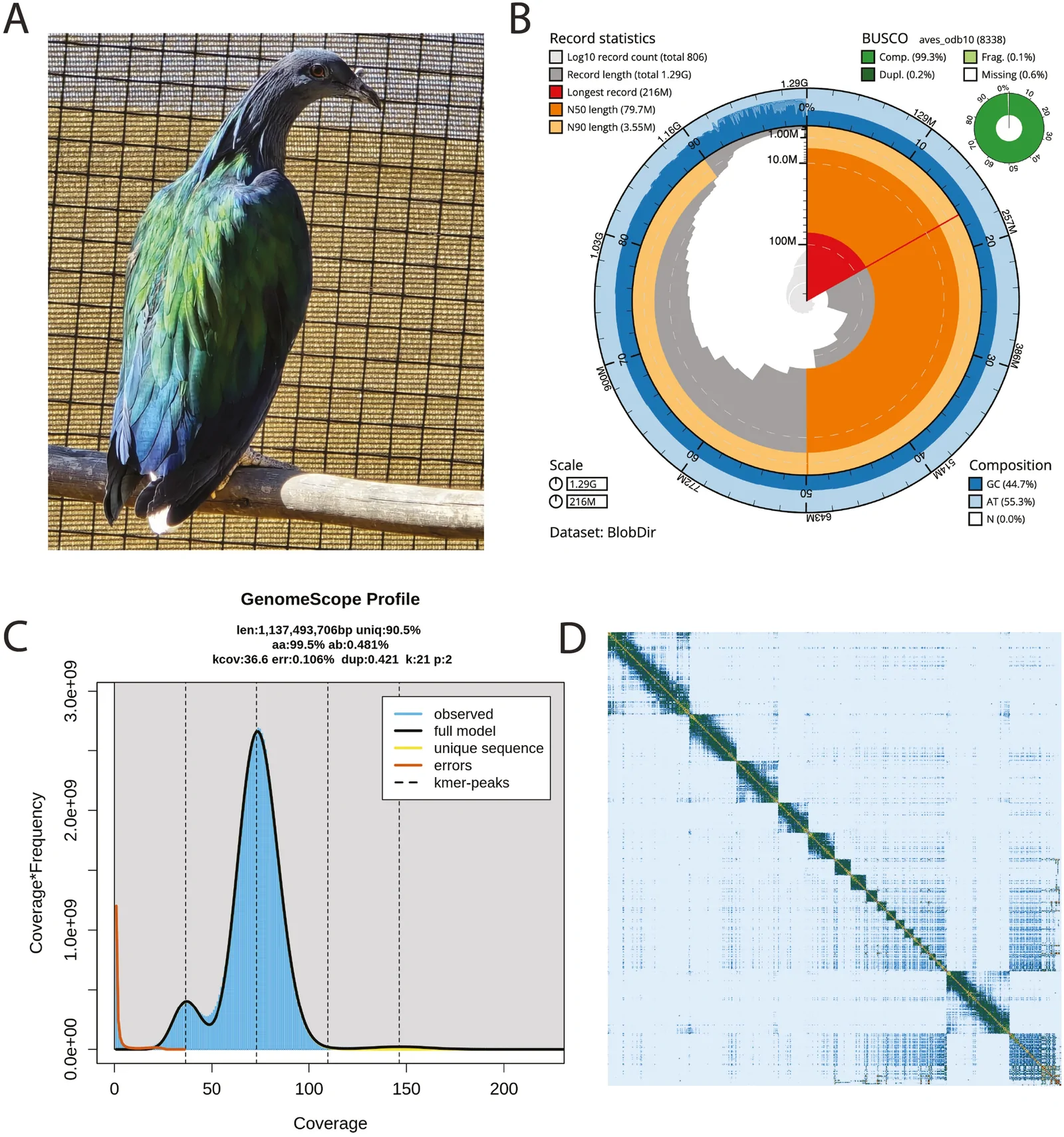
Nicobar pigeon photography and visual overview of genome assembly metrics. A) Photography of a Nicobar pigeon individual. B) BlobToolKit Snail plot illustrating quality metrics described in Table 2. C) K-mer spectra output generated with GenomeScope 2.0 from PacBio Hifi data showing genome size and heterozygosity estimates. D) Hi-C contact map generated with PretextView for the scaffolds representing the 40 chromosomes of bCalNic1.hap1.

We generated 85.4 Gb of PacBio HiFi data with an average read length of 16,153 bp and a median base quality of Q35. The k-mer spectral analysis revealed the two expected heterozygous and homozygous peaks at ~35- and ~70-fold coverage, respectively. We estimated a sequencing error rate of 0.106% and a nucleotide heterozygosity rate of ~0.48%, with an estimated genome size of 1.137 Gb (Fig. 1, Supplementary Table S1). Additionally, we produced 114.95 Gb of Illumina data for the Hi-C library, representing approximately ~88-fold coverage.

The final assembly, generated from the PacBio and Hi-C data, is composed of two haplotypes: bCalNic1.hap1 and bCalNic1.hap2. Analyses are reported for the bCalNic1.hap1 assembly, which is the primary haplotype, consisting of 923 contigs and 806 scaffolds, 117 gaps (20,400 bp total), with a total length of 1,285,834,579 bp. This assembly is highly contiguous, with a contig N50 of 25.1 Mb and a scaffold N50 of 79.7 Mb. The longest contig and scaffold are 97.7 Mb and 216.4 Mb, respectively. The bCalNic1.hap2 assembly is comparable, albeit more contiguous, but slightly shorter, with 344 contigs and 226 scaffolds, 118 gaps (20,600 bp total), totaling 1,197,404,482 bp. It has a contig N50 of 24.7 Mb, a scaffold N50 of 107.9 Mb, the longest contig is 96.1 Mb, and the longest scaffold is 215.7 Mb (Table 2, Supplementary Table S2).

**Table 2.** Genome completeness metrics for both haplotypes based on output from gfastats, Merqury, BUSCO.

Measure	bCalNic1.hap1	bCalNic1.hap2
Total length	1,285,834,579 bp	1,197,404,482 bp
Number of scaffolds	806	226
Scaffold L50/N50	5 scaffolds; 79.7 Mb	4 scaffolds; 107.9 Mb
Largest scaffold	216,431,472	215,715,690 bp
Number of contigs	923	344
Contig L50/N50	14 contig; 25.1 Mb	15 contigs; 24.7 Mb
Largest contig	97,706,909 bp	96,194,259 bp
Merqury completeness (both: 99.9169%)	92.5256%	92.5061%
Merqury QV (both: 66.0173)	65.0706	67.3416
BUSCO completeness	99.3%	99.3%
Single copy	99.1%	99.1%
Duplicated	0.2%	0.2%
Fragmented	0.1%	0.1%
Missing	0.6%	0.6%
Total	8338	8338

The completeness of the assembly and quality are high for both haplotypes (Supplementary Fig. S1). Using Merqury, k-mer completeness was estimated at 99.91% with a consensus quality value (QV) of ~66 for both haplotype1 and haplotype2 (Supplementary Tables S3 and S4). We have also estimated completeness with a Benchmarking Universal Single-Copy Orthologs (BUSCO) score of 99.3% ([Single copy: 99.1%, Duplicated: 0.2%], Fragmented: 0.1%, Missing: 0.6%) using the avian gene set aves_odb10 (Supplementary Table S5).

**Fig. 2.**
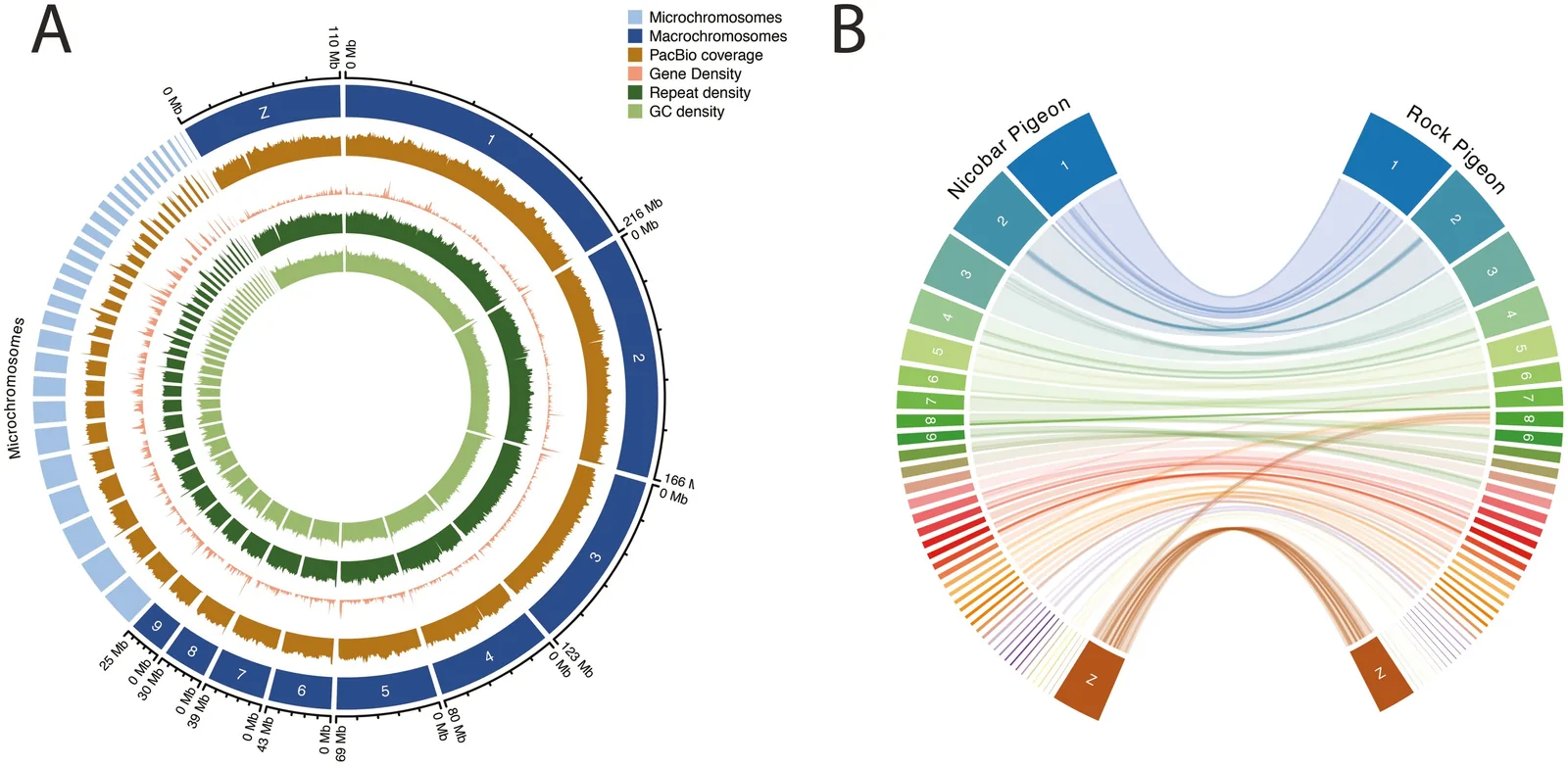
Nicobar pigeon assembly characteristics and synteny. A) Circos plot showing coverage, gene density, repeat density, and G-C content for the 40 chromosomes of bCalNic1.hap1. B) Chromosomes alignment of Nicobar pigeon (left) and rock pigeon (right) primary haplotype assemblies. Fragments of at least 500 kb are represented with a ribbon plot using Circos.

We identified 40 chromosomes in the bCalNic1.hap1 assembly, including the Z chromosome (Fig. 1), in agreement with previous karyotype studies (Fig. 2) (Belterman and De Boer 1984; Kiazim et al. 2021). Microchromosomes, characterized by a size of 20 Mb or less, constituted 30 of the assembled chromosomes.

We generated transcriptomic data for the muscle tissue to assist with genome annotation. These data include 3,505,709 PacBio Iso-Seq full-length reads (SRX24161358) and 174.5 M RNA-seq reads (SRX24161357, SRX24127190). bCalNic1.hap1 was annotated by the National Center for Biotechnology Information (NCBI) pipeline (GCF_036013445.1), resulting in the annotation of 7,126 coding genes and 1,462 noncoding genes, including 34,814 transcripts and 32,079 Coding DNA Sequence (CDSs).

## Discussion

To date, there are only 20 representative genome assemblies for Columbidae, and among them, only 10 are at the chromosome level, representing four species (*C. livia*, *Patagioenas fasciata*, *Streptopelia turtur*, *Nesoenas mayeri*). All four species belong to the Columbini tribe, part of the Holarctic-New World clade (Soares et al. 2016). The Nicobar pigeon assembly we present here is the first chromosome-level assembly representing the Indo-Pacific clade. Including genomes from both major Columbiformes clades in comparative genomics analyses will add significant value to our understanding of Columbiformes evolution and provide insights into how pigeons and doves have diversified and adapted to various habitats across the globe.

The Nicobar pigeon is the last remaining member of *Caloenas*. The spotted green pigeon (*Caloenas maculata*) was declared extinct sometime in the 1820s, and the Kanaka pigeon (*Caloenas canacorum*) is known only from subfossil remains (Hume 2017). The Nicobar pigeon also belongs to the Raphini tribe, which includes the Dodo and Rodrigues Solitaire, thus providing a unique opportunity to study the genomics of these extinct species. Given the close proximity of the Nicobar to multiple other large-bodied pigeon species, this Nicobar genome assembly will also serve as an important resource for studying the Island Effect in avian species (Clegg and Owens 2002; Losos and Ricklefs 2009; Wright et al. 2016).

Nicobar pigeons have a broad distribution in the Indo-Pacific spanning from the Andaman and Nicobar islands through the Indonesian archipelago and Solomon islands and are currently recognized as two subspecies, *C. nicobarica nicobarensis* and *C. nicobarica pelewensis*, the latter of which having a more restricted distribution near the Palau archipelago. As such, they represent a potential model for studies of island biogeography by enabling comprehensive genomic analyses to understand how isolated environments influence genetic diversity, adaptation, and speciation in birds. Additionally, this genome assembly can be used to further study the conservation of island populations and conservation strategies by identifying population structures, bottlenecks, and adaptive traits, which will aid in prioritizing habitat preservation and guiding breeding programs.

## Supplementary Material

esaf031_suppl_Supplementary_Tables_S1-S5

## Data Availability

The genome assembly (both haplotypes) and mitochondrial DNA sequence are available under NCBI BioProject PRJNA1061158 and PRJNA1061159, biosample SAMN39256628. This project is part of the Vertebrate Genomes Project PRJNA1061556. Raw genomic data are available on the GenomeArk repository (https://www.genomeark.org/genomeark-all/Caloenas_nicobarica.html) and transcriptome data are available in the Sequence Read Archive (SRA): accessions SRX24161358, SRX24161357, and SRX2412719). Mitochondrial genome: CM069764.
