# Supplementary material for: A chromosome-level genome of the Nicobar pigeon, Caloenas nicobarica
Source: J Hered. 2025 May 15;117(5):1144–9. doi: 10.1093/jhered/esaf031 (PMC13539444; doi:10.1093/jhered/esaf031)

Supplementary material

Supplementary Table 1. Summary of output from Genomescope 2.0 analysis using the following parameters: p=3; k=21; initial kmercov estimate = 36; TESTING set to TRUE.

property	min	max
Homozygous (aa)	99.5132%	99.5249%
Heterozygous (ab)	0.475067%	0.486838%
Genome Haploid Length	1,136,761,590 bp	1,137,493,705 bp
Genome Repeat Length	108,157,847 bp	108,227,505 bp
Genome Unique Length	1,028,603,742 bp	1,029,266,201 bp
Model Fit	88.1003%	95.8106%
Read Error Rate	0.106057%	0.106057%

Supplementary Table 2. Summary of output from GFAstats for both assembly haplotypes.

	bCalNic1.hap1	bCalNic1.hap2
# scaffolds	806	226
Total scaffold length	1,285,834,579	1,197,404,482
Average scaffold length	1,595,328.26	5,298,249.92
Scaffold N50	79,699,707	107,988,563
Scaffold auN	94,659,387.09	101,179,628.86
Scaffold L50	5	4
Largest scaffold	216,431,472	215,715,690
Smallest scaffold	16,521	15,175
# contigs	923	344
Total contig length	12,8581,4179	119,7383,882
Average contig length	1,393,081.45	3,480,767.10
Contig N50	25,101,800	24,693,610
Contig auN	33,378,896.52	31,940,676.21
Contig L50	14	15
Largest contig	97,706,909	96,194,259
Smallest contig	15010	15,175
# gaps in scaffolds	117	118
Total gap length in scaffolds	20,400	20,600
Average gap length in scaffolds	174.36	174.58
Gap N50 in scaffolds	200	200
Gap auN in scaffolds	185.29	185.44
Gap L50 in scaffolds	51	52
Largest gap in scaffolds	200	200
Smallest gap in scaffolds	100	100
Base composition (A:C:G:T)	359,557,279:287,971,523:286,010,012:352,275,365	341,930,826:257,378,830:256,803,612:341,270,614
GC content %	44.64	42.94
# soft-masked bases	0	0

# segments	923	344
Total segment length	1,285,814,179	1,197,383,882
Average segment length	1,393,081.45	3,480,767.10
# gaps	117	118
# paths	806	226

Supplementary Table 3. Output summary of Merqury with QV estimates.

assembly	unique k-mers	common k-mers	QV	error rate
bCalNic1.hap1	8421	1,288,855,765	65.0706	3.1113e-07
bCalNic1.hap2	4626	1,194,386,090	67.3416	1.84435e-07
both	13,047	2,483,241,855	66.0173	2.50192e-07

Supplementary Table 4. Output summary of Merqury for genome completeness.

assembly	k-mer set	solid k-mers	Solid k-mers in assembly	completeness %
bCalNic1.hap1	all	1,068,176,633	1,154,466,384	92.5256
bCalNic1.hap2	all	1,067,951,875	1,154,466,384	92.5061
both	all	1,153,507,483	1,154,466,384	99.9169

Supplementary Table 5. Output summary from BUSCO v.5.7.1 using lineage dataset aves_odb10. Creation date was 2024-01-08 using 62 genomes and 8338 BUSCOs in mode:euk_genome_min and the minprot gene predictor.

**bCalNic1.hap1**	
C:99.3%[S:99.1%,D:0.2%],F:0.1%,M:0.6%,n:8338,E:7.9%	
8279	Complete BUSCOs (C) (of which 658 contain internal stop codons)
8265	Complete and single-copy BUSCOs (S)
14	Complete and duplicated BUSCOs (D)
12	Fragmented BUSCOs (F)
47	Missing BUSCOs (M)
8338	Total BUSCO groups searched
**bCalNic1.hap2**	
C:99.3%[S:99.1%,D:0.2%],F:0.1%,M:0.6%,n:8338,E:8.0%	
8282	Complete BUSCOs (C) (of which 658 contain internal stop codons)
8267	Complete and single-copy BUSCOs (S)
15	Complete and duplicated BUSCOs (D)
12	Fragmented BUSCOs (F)
44	Missing BUSCOs (M)
8338	Total BUSCO groups searched

Supplementary Figure 1. Merqury kmer spectra plots for both haplotypes.

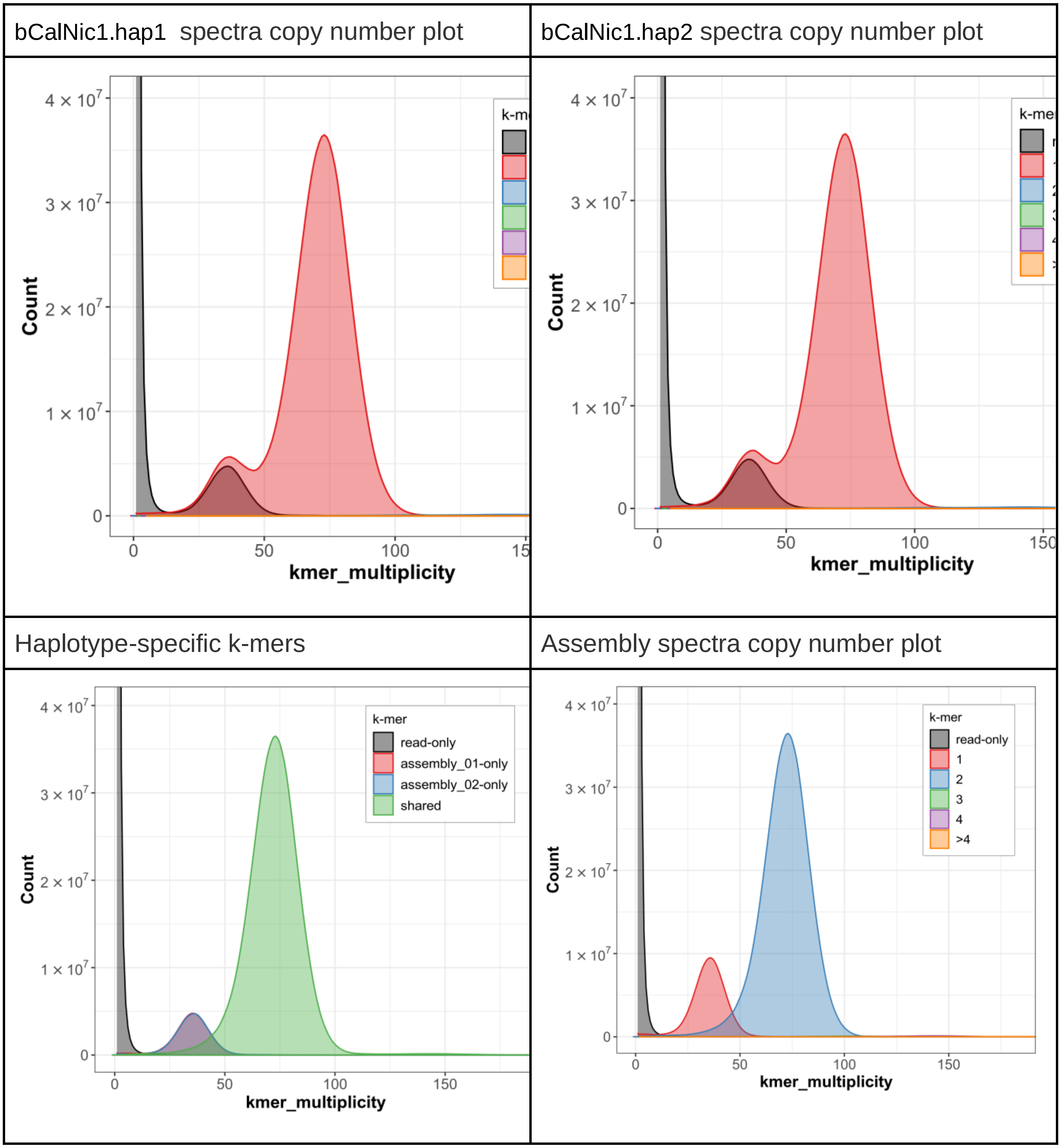

Supplement: Supplementary file 1 [file esaf031_suppl_supplementary_tables_s1-s5.pdf]
